# Localisation of Lactate Transporters in Rat and Rabbit Placentae

**DOI:** 10.1155/2016/2084252

**Published:** 2016-10-23

**Authors:** Nigel P. Moore, Catherine A. Picut, Jeffrey H. Charlap

**Affiliations:** ^1^Ubrs GmbH, Postfach, 4058 Basel, Switzerland; ^2^WIL Research, LLC, Hillsborough, NC 27278, USA; ^3^WIL Research, LLC, Ashland, OH 44805, USA

## Abstract

The distribution of monocarboxylate transporter (MCT) isoforms 1 and 4, which mediate the plasmalemmal transport of l-lactic and pyruvic acids, has been identified in the placentae of rats and rabbits at different ages of gestation. Groups of three pregnant Sprague-Dawley rats and New Zealand White rabbits were sacrificed on gestation days (GD) 11, 14, 18, or 20 and on GD 13, 18, or 28, respectively. Placentae were removed and processed for immunohistochemical detection of MCT1 and MCT4. In the rat, staining for MCT1 was associated with lakes and blood vessels containing enucleated red blood cells (maternal vessels) while staining for MCT4 was associated with vessels containing nucleated red blood cells (embryofoetal vessels). In the rabbit, staining for MCT1 was associated with blood vessels containing nucleated red blood cells while staining for MCT4 was associated with vessels containing enucleated red blood cells. Strength of staining for MCT1 decreased during gestation in both species, but that for MCT4 was stronger than that for MCT1 and was consistent between gestation days. The results imply an opposite polarity of MCT1 and MCT4 across the trophoblast between rat and rabbit.

## 1. Introduction

Lactate transport is achieved by members of the monocarboxylate transporter (MCT) family, or SLC16 solute carrier family, a class of plasma membrane transport proteins. MCT isoforms 1–4 are symporters that mediate the proton-dependent transport of small monocarboxylic acids, particularly l-lactic acid; pyruvic acid; and the ketone bodies, acetoacetic (3-oxobutyric) acid and 3-hydroxybutyric acid [1, 2]. MCT1–4 have widely differing affinities for l-lactate and pyruvate. The different substrate affinities, as well as tissue distribution, between these four isoforms reflect their roles in energy metabolism. MCT1 and MCT2 are expressed in cells that use lactate as a respiratory fuel or for gluconeogenesis, while MCT3 and MCT4 are associated with lactate efflux from highly glycolytic cells, although MCT1 can also mediate lactate efflux under hypoxic conditions [3, 4].

Northern blot analysis of pooled human placenta (Clontech human tissue panels) has identified mRNA for MCT1 and MCT4, but not MCT2 [5, 6]; while early work reported the presence of MCT3 mRNA or protein expression [6, 7], this isoform was later reclassified as MCT4 [3, 6]. Subsequent Western blot analysis of individual human term placentae showed expression of both MCT1 and MCT4 [7–9], although the distributions of the two isoforms within the syncytiotrophoblast differ. While MCT1 is localised predominantly towards the basal plasma membrane opposed to the foetal blood, MCT4 is localised predominantly towards the maternal-facing microvillous plasma membrane. They are not strictly segregated, however, and there is some degree of coincidence [9–11]. In contrast, while both isoforms are found in the mouse placenta, their polarity is the opposite of that in human placenta. Specifically, MCT1 is localised predominantly on the apical side of the syncytiotrophoblast I, adjacent to the maternal blood, and MCT4 is found on the basal side of the syncytiotrophoblast II, adjacent to the foetal blood [10].

Beyond these findings in human and mouse placenta, nothing is known of the distribution of lactate transporters in the placenta. The purpose of the studies reported here was to investigate the localisation of MCT1 and MCT4 in the placentae of two species that are commonly used in reproduction toxicity studies, the rat and the rabbit.

## 2. Materials and Methods

### 2.1. Animals

The animal facilities at WIL Research are fully accredited by AAALAC International, and all maintenance and experimental procedures were conducted in compliance with National Research Council guidelines [12]. All procedures were conducted according to Good Laboratory Practice.

Sexually mature, virgin female Crl:CD(SD) rats (Charles River Laboratories, Inc., Kingston, NY) and time-mated female New Zealand White Hra:(NZW)SPF rabbits (Covance Research Products, Inc., Greenfield, IN) were received in good health from the breeders. Rats were approximately eighty days old upon receipt; rabbits were approximately six months old and were received on GD 1. Each animal was examined on the day of receipt and uniquely identified by ear tag.

### 2.2. Animal Procedures

All animals were kept in environmentally controlled rooms with a twelve-hour light/dark photoperiod, maintained at a temperature of 22 ± 3°C (rats) or 19 ± 3°C (rabbits), and relative humidity of 50 ± 20%. Upon arrival and until pairing, all rats were housed individually in clean, stainless steel, wire-mesh cages suspended above cage-board that was changed at least thrice weekly. The rats were paired for mating in the home cage of the male. Following positive evidence of mating, designated GD 0, the females were returned to individual suspended wire-mesh cages. Rabbits were housed individually in clean, stainless steel cages suspended above ground corncob bedding which was changed twice weekly. For the duration of the study, animals were maintained on Certified Rodent LabDiet® 5002 or Certified Rabbit LabDiet® 5322 (PMI Nutrition International, St. Louis, MO); rabbits were also provided kale leaf.

Groups of three rats were euthanised by carbon dioxide inhalation on GD 11, 14, 18, or 20. Groups of three rabbits were euthanised by an intravenous injection of sodium pentobarbital via a marginal ear vein on GD 13, 18, or 28. The thoracic, abdominal, and pelvic cavities were opened by a ventral mid-line incision, and the contents were examined. The uterus was exposed, excised, and trimmed. Placentae were collected from three embryos or foetuses from each gestation day and retained in 10% neutral-buffered formalin. Viable foetuses were euthanised by a subcutaneous injection of sodium pentobarbital in the scapular region.

### 2.3. Immunohistochemistry

Following fixation for 48–72 hours, placentae were transferred to 70% ethanol. The tissues were trimmed and processed into paraffin blocks, sectioned, and mounted on glass microscope slides.

*MCT1 Staining*. Slides were stained immunohistochemically using the Ventana Discovery XT automated slide staining system (Ventana Medical Systems Inc., Tucson, AZ). The Ventana DabMap detection system was used. Antigen retrieval was obtained using CC1 (cell conditioning solution, pH 8.0). The primary antibody was chicken anti-rat MCT1 (EMD Millipore, Billerica, MA; catalogue number AB1286-I), and the secondary antibody was donkey anti-chicken IgY (Jackson Immunoresearch, reference number 703-065-155).

*MCT4 Staining*. Antigen retrieval was obtained in a DIVA decloaker at full power for two 5-minute cycles. Background staining was blocked by two procedures, using hydrogen peroxide and the Stirrup blocking solution. Slides were stained immunohistochemically using the Ventana Discovery XT slide staining system with rabbit anti-human MCT4 (Biorbyt LLC, San Francisco, CA; catalogue number orb137272) as the primary antibody, and goat anti-rabbit as the secondary antibody (Jackson Immunoresearch, reference number 111-066-003). The avidin-biotin (ABC) detection method was used followed by diaminobenzidine (DAB) as the chromagen.

Slides were counterstained with haematoxylin. Qualitative microscopic examination, including determination of staining localisation and staining intensity on a scale of 1 to 4, was performed on the stained sections by a board-certified veterinary pathologist.

## 3. Results

### 3.1. Localisation of MCT in Rat Placenta

**Figure 1:**
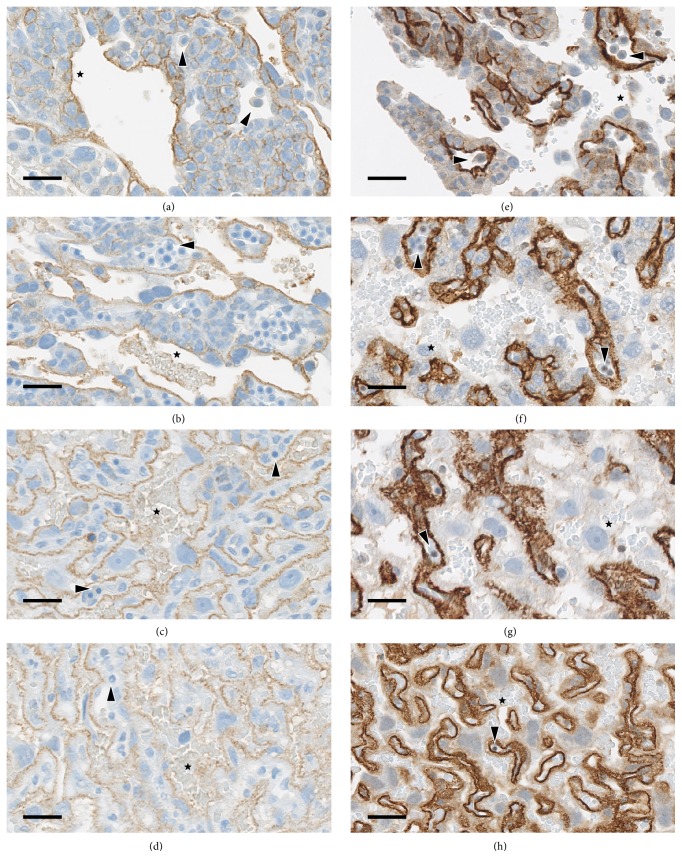
Localisation of MCT1 and MCT4 in rat placenta at four different ages during gestation. The staining of rat placenta with antibodies to (a–d) MCT1 and (e–h) MCT4 is shown for (a, e) GD 11; (b, f) GD 14; (c, g) GD 18; and (d, h) GD 20. Maternal blood vessels and cells are indicated with stars (★) and foetal blood vessels and cells are indicated with arrow heads (▸). Original objective 40x, bar 15 *μ*m.

Staining for both MCT1 and MCT4 in rat placentae on GD 11, 14, 18, and 20 was limited to the labyrinth zone. Within the labyrinth, MCT1 was present on the maternal side of the trophoblasts (Figures 1(a)–1(d)). The staining was moderate and formed a thick line outlining the lakes or vessels that were filled with enucleated red cells (i.e., the maternal vessels) and in many cases existed circumferentially around the trophoblasts forming a “chicken-wire” pattern. This moderately intense specific staining of the maternal side of trophoblasts, with nonstaining of the foetal side, was most apparent at GD 14 where there was a significant number of foetal vessels that were distinguished by foetal nucleated red cells and surrounded by unstained syncytiotrophoblasts. There was linear moderate staining of cytotrophoblasts at edge of maternal vessels (Figure 1(b)). At GD 11, there was a limited amount of labyrinth present, and foetal vessels were not readily identified in the deep labyrinth. There was moderate staining for MCT1 in cytotrophoblasts bordering maternal vessels, but no staining was present immediately around the foetal blood vessels (Figure 1(a)). Specific staining for MCT1 was less intense (mild) at the periphery of maternal vessels in the GD 18 and GD 20 placentae, when compared to GD 11 and GD 14, and there was nonspecific background staining of maternal, enucleated, red cells (Figures 1(c) and 1(d)). Few nucleated foetal red cells, surrounded by unstained trophoblasts, were identified in the GD 18 placentae and were rarely identified in the GD 20 placentae.

Staining for MCT4 in the labyrinth zone of the placenta was uniformly and very strongly positive (Table 1) and was limited to the foetal side of the syncytiotrophoblasts (Figures 1(e)–1(h)), forming a thick line outlining foetal blood vessels, which were characterised by nucleated foetal erythrocytes. Cells with larger nuclei were unstained cytotrophoblasts that border the maternal blood vessels which contain enucleated erythrocytes. The cellular location of the stain (i.e., whether basement membrane or cell membrane) could not be determined by light microscopy. Nucleated red cells were apparent within the lumina of foetal blood vessels at GD 11, 14, and 18 but were few at GD 18 and were exceedingly rare at GD 20.

There was occasionally cell surface staining of glycogen cells of the giant cell layer and of the decidua; however, this staining was far less intense than that of syncytiotrophoblasts and was considered to represent nonspecific background staining.

### 3.2. Localisation of MCT in Rabbit Placenta

**Figure 2:**
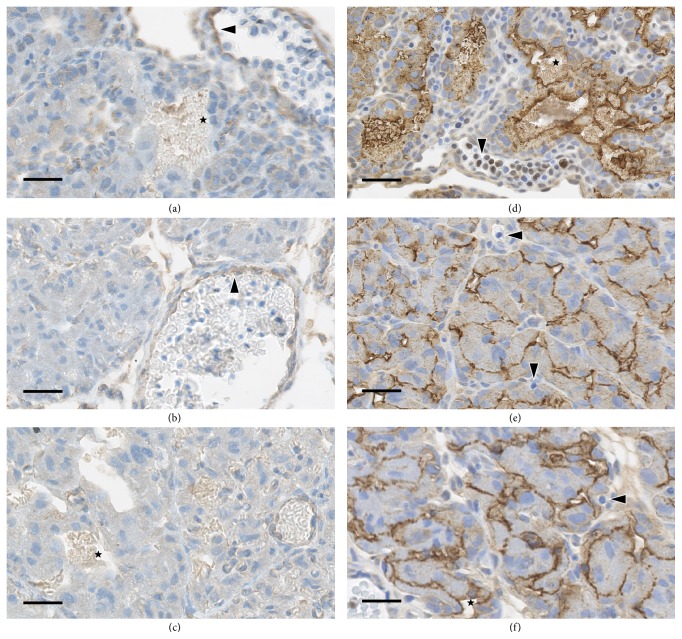
Localisation of MCT1 and MCT4 in rabbit placenta at three different ages during gestation. The staining of rabbit placenta with antibodies to (a–c) MCT1 and (d–f) MCT4 is shown for (a, d) GD 13; (b, e) GD 18; and (c, f) GD 28. Maternal blood vessels and cells are indicated with stars (★) and foetal blood vessels and cells are indicated with arrow heads (▸). Original objective 40x, bar 15 *μ*m.

There was mild staining of MCT1 along the lining of blood vessels containing nucleated foetal red cells at GD 13 (Figure 2(a)), which was reduced to minimal intensity at GD 18 (Figure 2(b)). These positive vessels were located at the periphery of the chorion and labyrinth. At GD 28, there was no specific positive staining of trophoblasts surrounding maternal red blood cells, and there were no vessels containing foetal red blood cells (Figure 2(c)). There was no specific positive staining for MCT1 within the body of the labyrinth at any stage of gestation. Both vessels and lakes filled with either maternal or foetal red cells were lined by syncytiotrophoblasts that had no surface staining for MCT1. There was slight nonspecific (i.e., background) staining of maternal red cells.

Staining for MCT4 was strong on GD 13, 18, and 28 and limited to the maternal side of the syncytiotrophoblasts (Figures 2(d)–2(f)). At GD 13 and 18, the positive staining formed a thick line outlining the maternal blood vessels throughout the entire labyrinth. At GD 28, there was more extensive staining of the cytoplasmic membrane of the syncytiotrophoblasts, where the staining was not limited to the side of the cell facing the lumen of the vessel. This more expansive staining resulted in a “chicken-wire” appearance. Nucleated foetal red cells are no longer present in the placenta at this stage of gestation. There was no staining along trophoblasts lining the foetal vessels at any stage.

There was nonspecific cell surface staining of glycogen cells of the giant cell layer and of the decidua and light background staining of maternal red blood cells.

## 4. Discussion

By using antibodies against the C-termini of MCT1 (chicken anti-rat) and MCT4 (rabbit anti-human), both isoforms have been identified in the placenta of rat and rabbit at different days of gestation. MCT1 was predominantly localised to the maternal side of the trophoblast in the rat, but to the foetal side in the rabbit. Conversely, MCT4 was localised towards the foetal side in the rat and the maternal side in the rabbit. The localisation of the two isoforms in the rabbit is similar to that previously reported in human placenta [9–11]; localisation in the rat is similar to that previously reported in mouse placenta [10].

The polarity remained constant throughout gestation in both species, as has also been reported for the mouse [10]. MCT1 is also localised at the basal membrane in the four-month old human placenta as well as the term placenta [10], which indicates that its distribution, at least in qualitative terms, also may not change during gestation.

The staining for both isoforms was generally stronger in the rat than in the rabbit at equivalent stages of gestation (Table 1). In both species, the expression of MCT1, as evidenced by strength of staining, appeared to decrease during gestation, while that of MCT4 remained consistent. The expression of both MCT1 and MCT4 mRNA is reported to diminish during later gestation, in contrast to that of the type 1 glucose transporter the expression of which was consistently intense [10].

Both MCT1 and MCT4 have a low degree of substrate-specificity, transferring a relatively wide range of substituted and unsubstituted monocarboxylic acids across the plasma membrane [13–16]. The major difference between the two isoforms is their substrate affinity. The affinity of MCT1 towards l-lactate and pyruvate is relatively high, within the normal physiological range for blood concentrations [13, 14], while that of MCT4 is relative low [15, 16]. These differences are postulated to underlie the function of the two isoforms, MCT1 sequestering lactate as a source for energy metabolism and growth and MCT4 releasing lactate during periods of high cellular production [1, 3, 4].

It follows, therefore, that human and rabbit trophoblasts have a high-affinity (MCT1) transporter on the foetal side and a low-affinity (MCT4) transporter on the maternal side, while murine trophoblasts have a low-affinity (MCT4) transporter on the foetal side and a high-affinity (MCT1) transporter on the maternal side. The MCT is a proton-sensitive symporter, and the rate and direction of plasmalemmal transfer will be determined not only by the concentration gradient of the monocarboxylate anion but also by the proton gradient, which may change during gestation as the foetus becomes net lactogenic and the foetal blood becomes acidic in comparison to that of the maternal blood towards the end of gestation.

In conclusion, there is a difference in the localisation of MCT1 and MCT4 between the murine placenta on the one hand and the rabbit and human placentae on the other. Essentially, the “polarity” of these isoforms across the trophoblast is reversed. The functional significance of this difference is unclear, although it has been postulated that it is relevant to the direction of substrate transport [10]. It may also reflect differences in the role of the trophoblast in lactate supply, either by transfer from the maternal blood or by metabolism of glucose or amino acids, and the disposition of maternally derived 2- and 3-hydroxyl- or carbonyl-substituted carboxylic acids that are also MCT substrates. Given the complexity of monocarboxylic acid transport across the trophoblast, further data from specifically designed, integrated studies are required to elucidate the functional significance of the differences in MCT isoform localisation and changes in expression during gestation.

## Figures and Tables

**Table 1:** Strength of staining for MCT isoforms 1 and 4 in rat and rabbit placentae at different days of gestation.

Species	GD	MCT1	MCT4
Rat	11	3	4+
	14	3	4+
	18	2	4+
	20	2	4+
Rabbit	13	2	4
	18	1	4
	28	0	4

Graded staining intensity: 0, none; 1, minimal; 2, mild; 3, moderate; 4, strong; 4+, very strong.

## Abbreviations

GD: Gestation day(s)
MCT: Monocarboxylate transporter.
